# Total talar replacement for metastatic pulmonary adenocarcinoma of the talus: A case report

**DOI:** 10.1002/ccr3.9049

**Published:** 2024-06-21

**Authors:** Tatsuya Sakai, Masanori Fujii, Masaaki Mawatari

**Affiliations:** ^1^ Department of Orthopaedic Surgery, Faculty of Medicine Saga University Saga Japan

**Keywords:** case report, metastatic bone tumor, pulmonary adenocarcinoma, talar replacement, talus

## Abstract

Talar metastases from malignant tumors are rare and poorly documented. Treatment requires gradual relief of pain and preservation of function, with a choice between palliative measures and surgery. This case indicates that total talar replacement is an effective intervention for localized talar metastases and highlights the importance of early intervention. A 48‐year‐old man was diagnosed with a pathologic talar fracture due to talar metastases was observed after 8 years of chemotherapy following a diagnosis of lung adenocarcinoma. Despite radiotherapy, the patient's activities of daily living (ADLs) deteriorated due to pain on walking, prompting a request for surgical intervention. Total talar replacement was performed, allowing the patient to begin full weight‐bearing ambulation 2 weeks post‐operatively. Total talar replacement appears to be an effective treatment for localized talar metastases and should be performed as early as possible.

## 1 INTRODUCTION

Metastatic bone tumors are more common than primary tumors. However, bone metastases to the foot and ankle from primary malignancies are very rare, reported in 0.003%–0.01% of cancer patients.^1^, ^2^ Few reports exist about talar metastases, and the mechanism by which tumor cells metastasize to the foot is not yet fully understood.

Bone metastases are mainly treated with radiotherapy and chemotherapy; however, if the lesions are osteolytic and located in the weight‐bearing zone, pain is unlikely to improve, and activities of daily living (ADLs) are significantly impaired. Few reports on the surgical treatment for these patients have been published and no reports exist about a reliable method to slow pain and promote early ADL improvement.^3^, ^4^, ^5^

Herein, we describe a case of pathological fracture after talar metastasis from lung cancer, in which artificial talar replacement was performed and the bility to perform ADLs improved.

## 2 CASE PRESENTATION

**FIGURE 1:**
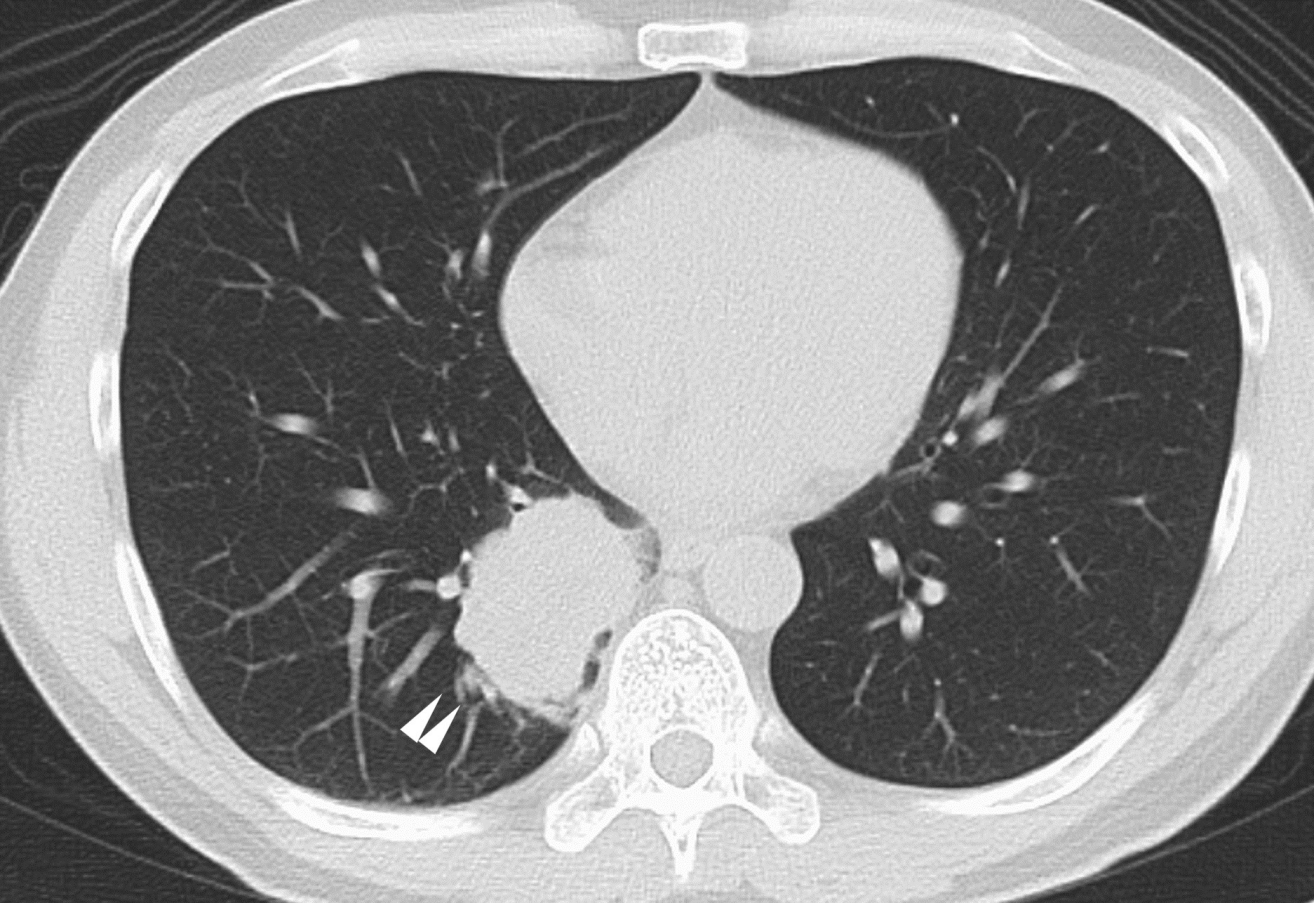
Chest computed tomography for the diagnosis of lung cancer. The white arrow points to a 53 × 39 mm lesion.

A 48‐year‐old man was diagnosed with adenocarcinoma of the right lung with brain metastases 8 years prior (Figure 1). The patient received chemotherapy at our hospital, starting with epidermal growth factor receptor‐tyrosine kinase inhibitor (EGFR‐TKI) treatment, which was administered up to the eighth line of treatment for 8 years. Gamma Knife radiosurger was used for resection of a metastatic brain lesion 3 years after initial diagnosis. The primary tumor did not grow as a result of the chemotherapy, but a tendency to grow was observed 7 years after the initial diagnosis. Moreover, compared to levels at the time of primary tumor diagnosis (CEA; 19.6 ng/mL and CYFRA; 7.19 ng/mL), tumor markers were also elevated 7 years after initial diagnosis, even though they had normalized following treatment of the primary tumor.

On one occasion, he developed pain in his right ankle when jumping, and a magnetic resonance imaging (MRI) scan 1 month after the onset of symptoms revealed a pathological fracture of the talus. Close examination revealed pleural dissemination and bilateral iliac metastases. The patient was referred to our department for treatment of the right ankle.

Upon physical examination, the patient was wheelchair‐bound outdoors and ambulated indoors with a walker. The brain metastases resulted in paralysis of the left upper and lower limbs. The right ankle was painful on exertion and at rest, with a numerical rating scale (NRS) of 9–10. The range of motion was preserved, and The Eastern Cooperative Oncology Group performance status (PS) was 2. The American Orthopaedic Foot and Ankle Society (AOFAS) ankle/hind foot score was 62.

**FIGURE 2:**
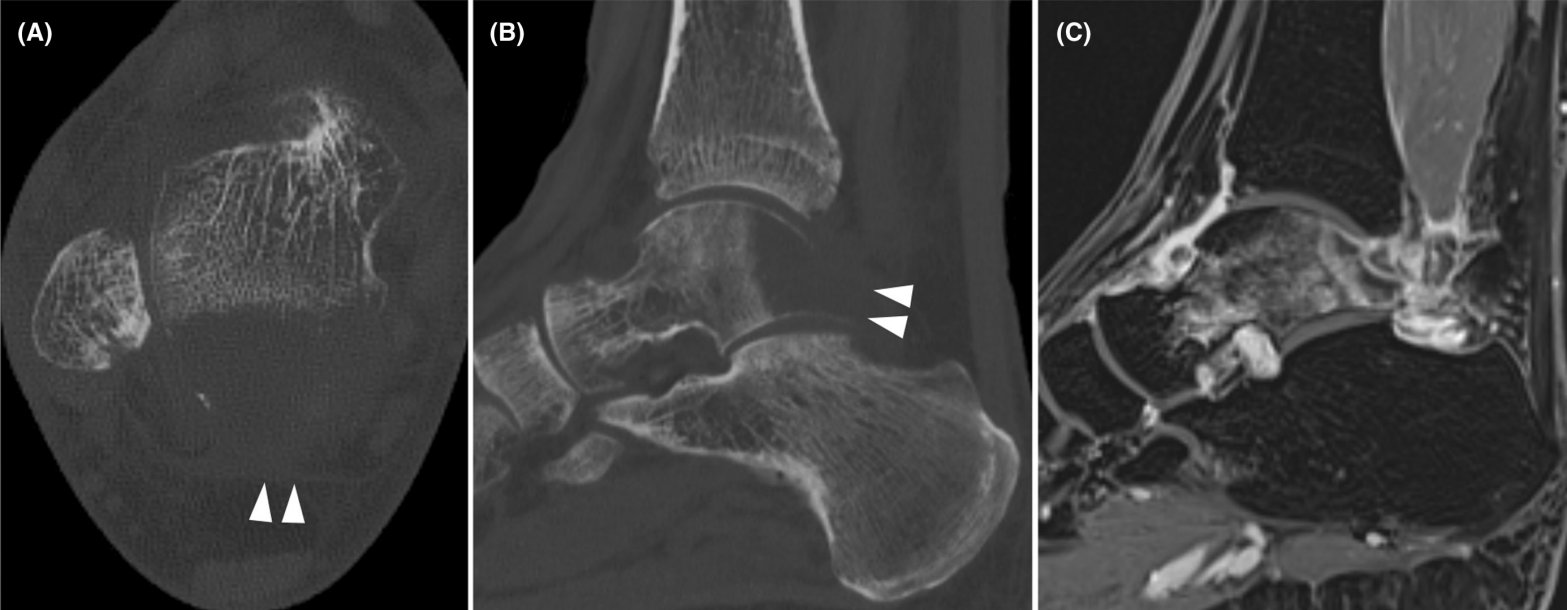
Preoperative ankle computed tomography: axial (A) and sagittal (B). The white arrows indicate the osteolytic lesion. Preoperative ankle magnetic resonance imaging: sagittal (C).

Standing plain radiographs showed osteolytic changes in the posterior process of the talus in the lateral view. Computed tomography (CT) showed osteolytic changes extending into the body of the talus (Figure 2A,B). MRI showed a well‐defined abnormal signal in the posterior process and a slight extraosseous extension. Edema was observed in the periosteal marrow of the body, along with intra‐articular fluid retention and synovial enhancement (Figure 2C).

## 3 METHOD

Based on the imaging findings, the patient was diagnosed with a pathological fracture of the talus following talar metastasis of the lung adenocarcinoma. According to the department that was treating the patient, 8 years had passed since the onset of lung cancer and brain metastases, and the prognosis was inconclusive; therefore, radiotherapy and bone‐modifying drugs were initiated. A brace was initially considered for persistent weight‐bearing pain but was impractical and difficult to use due to the paralysis of the left upper and lower extremities; therefore, surgery was considered for pain relief and ADL improvement.

**FIGURE 3:**
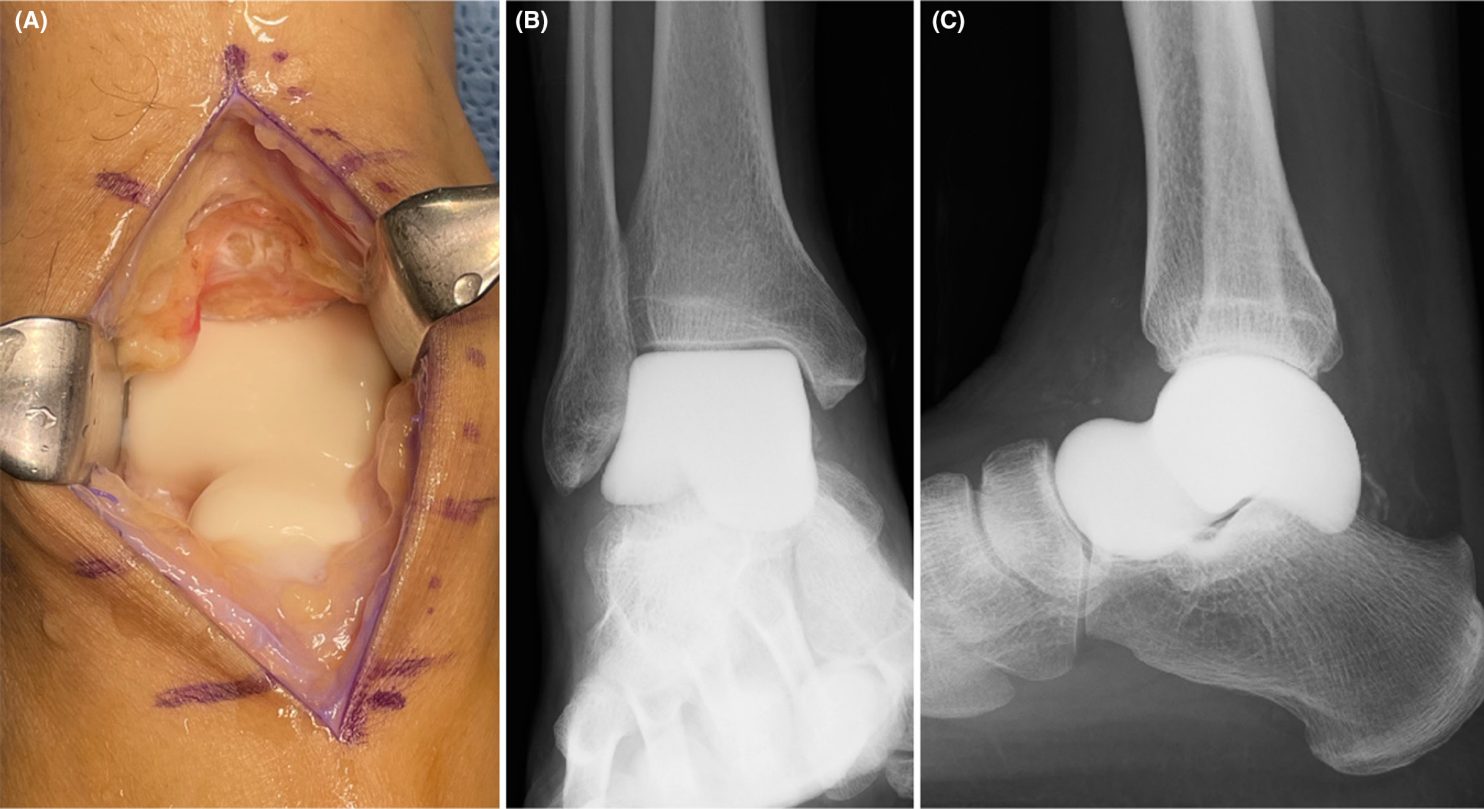
Intraoperative gross findings: after talar replacement (A). Postoperative radiographs of the ankle: frontal (B) and lateral (C).

Because the lesion was almost completely confined to the talus, total talar replacement was performed 2 months after diagnosis (Figure 3). After 2 weeks of external fixation and offloading, the patient was allowed to walk with a load based on pain intensity and was discharged home 3 weeks after surgery with the ability to stably walk with a cane.

**FIGURE 4:**
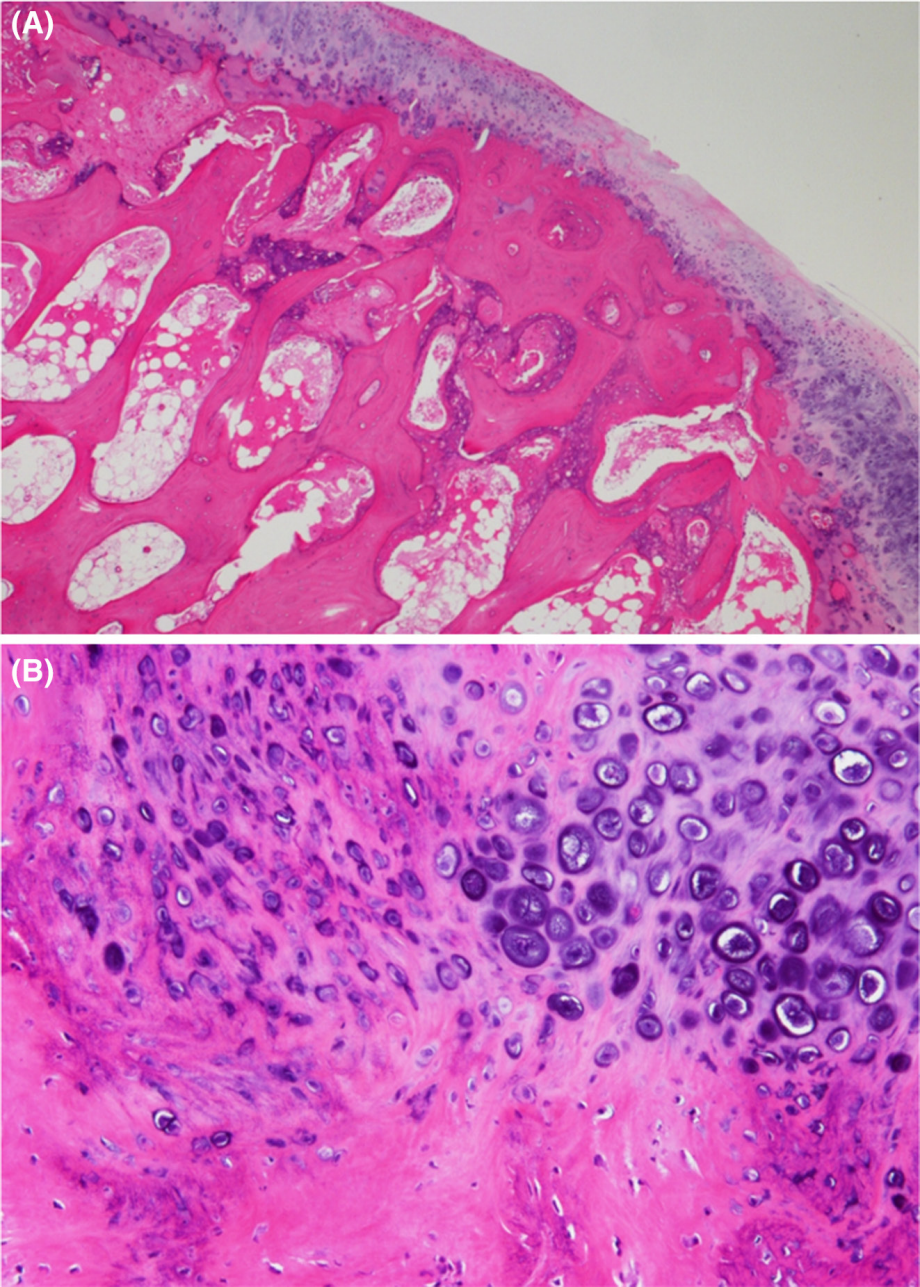
Postoperative histopathological photographs show neither obvious adenocarcinoma cells nor other malignant findings. (A) Hematoxylin and eosin stain at ×40 magnification. (B) Hematoxylin and eosin stain at ×400 magnification.

Pathology results showed no obvious adenocarcinoma cells or other malignant findings; however, ossification with osteoblasts, fibroblast proliferation, and fibrous bone formation were consistent with the findings after irradiation and pathological fracture (Figure 4).

## 4 CONCLUSION AND RESULTS

One year after surgery, the patient was able to walk with a cane indoors, and his right ankle joint was pain free (an NRS scale of 0), with an AOFAS score of 83, while the PS was maintained at 2. Additionally, MRI showed no new metastases or signal changes in the talipes; however, intra‐articular fluid retention and synovial strengthening effects improved. Thereafter, his general condition gradually deteriorated and he died 18 months after surgery.

Here, we report a case of artificial talar replacement for a pathological fracture following talar metastasis from lung adenocarcinoma. Artificial talar replacement is useful for tumors confined to the talus. However, selecting an appropriate treatment for an individual patient's condition and promptly administering it is important.

## 5 DISCUSSION

The reported incidences of site‐specific bone metastases in the foot were calcaneus, phalanges, metatarsals, and talus.^6^ The most common primary site of bone metastases to the foot is the lungs, but the most common site is the urological system, including the kidneys, bladder, and prostate. Isolated reports involving metastasis from the breast and colon also have been published.^6^ However, the mechanism by which tumor cells metastasize to the foot is not yet fully understood.

Treatment of metastatic bone tumors requires both pain and symptoms relief as well as motor function preservation and can be achieved by conservative or surgical management. We found three reports of metastatic talar tumors.^3^, ^4^, ^5^ The main treatment chosen was resection of the lesion and bone cement implantation^3^, ^5^; however, no reports exist which discuss methods of pain alleviation or promotion of early ADL extension.

In the present case, the metastases were almost entirely confined to the talus, and the patient was considered to have a good prognosis. Artificial talar replacement was performed 3 months after the pathological fracture. The artificial talus used in this study was a customized ceramic implant. A CT scan of the healthy side was performed, and the implant was created based on the images.^7^ Joint function was preserved, and no differences in leg length or early weight bearing were observed.

The pain was relieved immediately after surgery. Additionally, full weight‐bearing was started 2 weeks after surgery, and the patient was discharged after walking independently with a cane and was able to maintain his ADL until his general condition deteriorated. Artificial talar replacement is an effective treatment option for talar metastasis. No reports of artificial talar replacement for metastatic tumors have been published, and this is the first report of such a procedure for metastatic talar tumors.^8^, ^9^, ^10^

With advances in the treatment of malignant tumors, cancer survival rates are increasing in many areas, and metastatic bone tumors are also expected to increase. The mean time from the diagnosis of the primary tumor to the confirmation of metastasis to the foot was reported to be 93 months, while the mean time to accurate diagnosis was 6.4 months.^11^ In the present case, talar bone metastasis was detected 8 years after the diagnosis of lung adenocarcinoma.

Furthermore, reports suggest that distal extremities may have been excluded from the systemic assessment of metastases because the feet of patients with advanced metastatic disease who are unable to walk are often not considered.^6^ These factors suggest that bone metastases to the foot and ankle are rare and may be missed by clinicians. The occurrence of these metastases is likely to increase in the future. Therefore, accurate and timely diagnosis and treatment appropriate for the patient's condition, such as regular bone scintigraphy, should be offered and implemented.

## AUTHOR CONTRIBUTIONS

**Tatsuya Sakai:** Data curation; visualization; writing – original draft; writing – review and editing. **Masanori Fujii:** Conceptualization; methodology. **Masaaki Mawatari:** Supervision.

## FUNDING INFORMATION

The authors of this article declare that no funding was obtained for the writing and preparation of this manuscript.

## CONFLICT OF INTEREST STATEMENT

The authors declare that they have no conflicts of interest with any organization regarding the article submitted for publication.

## ETHICS STATEMENT

This involves the description of a clinical case. It is not a clinical trial, and no experimentation has been conducted on animals or humans. The authors of this manuscript have adhered to the protocols of our workplace for the publication of clinical cases, and patient anonymity has been preserved.

## CONSENT

Written informed consent was obtained from the patient to publish the current case report in accordance with the journal's patient consent policy.

## Data Availability

We commit to ensuring the availability and confidentiality of the information related to the presented clinical case. Our priority is to safeguard patient privacy and adhere to the highest ethical standards in handling medical information. All clinical documentation will be protected and used solely for educational and academic discussion purposes.
